# Identification and Quantification of Hydrocarbon Functional Groups in Gasoline Using ^1^H-NMR Spectroscopy for Property Prediction

**DOI:** 10.3390/molecules26226989

**Published:** 2021-11-19

**Authors:** Abdul Gani Abdul Jameel

**Affiliations:** 1 Department of Chemical Engineering, King Fahd University of Petroleum & Minerals, Dhahran 31261, Saudi Arabia; a.abduljameel@kfupm.edu.sa; 2 Center for Refining & Advanced Chemicals, King Fahd University of Petroleum & Minerals, Dhahran 31261, Saudi Arabia

**Keywords:** functional groups, ^1^H-NMR, octane number, cetane number, gasoline

## Abstract

Gasoline is one of the most important distillate fuels obtained from crude refining; it is mainly used as an automotive fuel to propel spark-ignited (SI) engines. It is a complex hydrocarbon fuel that is known to possess several hundred individual molecules of varying sizes and chemical classes. These large numbers of individual molecules can be assembled into a finite set of molecular moieties or functional groups that can independently represent the chemical composition. Identification and quantification of groups enables the prediction of many fuel properties that otherwise may be difficult and expensive to measure experimentally. In the present work, high resolution ^1^H nuclear magnetic resonance (NMR) spectroscopy, an advanced structure elucidation technique, was employed for the molecular characterization of a gasoline sample in order to analyze the functional groups. The chemical composition of the gasoline sample was then expressed using six hydrocarbon functional groups, as follows: paraffinic groups (CH, CH_2_ and CH_3_), naphthenic CH-CH_2_ groups and aromatic C-CH groups. The obtained functional groups were then used to predict a number of fuel properties, including research octane number (RON), motor octane number (MON), derived cetane number (DCN), threshold sooting index (TSI) and yield sooting index (YSI).

## 1. Introduction

Crude oil is one of the most chemically complex substances naturally present, and a drop of it is known to contain several hundred thousands of individual molecules of various sizes, structure and functionalities [1,2]. Fractional distillation of petroleum crude produces various distillate and residual fuels that are used for a variety of purposes including transportation, power generation, heating, etc. [3,4,5]. Gasoline is one of the highly volatile cuts obtained at a temperature range between 30 °C to 200 °C, and is predominantly used as a transportation fuel in SI engines. The molecular structure of a petroleum fuel defines its combustion chemistry, and its molecular characterization is thus the first step in understanding the fuel’s combustion behavior, one that also helps in property prediction. Detailed hydrocarbon analysis (DHA), which identifies and quantifies the individual molecules in gasoline range fuels, has shown that a series of standard gasolines referred to as FACE (fuels for advanced combustion engines) gasolines contain around a hundred individual molecules [6]. Knowledge of the fuel’s chemical composition is needed in order to develop fuel surrogates, which are usually a mixture of two or more species that aim to reproduce the fuel’s physical, thermochemical and combustion behavior [7]. Based on the chosen surrogate species, detailed chemical kinetic models are developed and used to understand combustion phenomena such as ignition delay time (IDT), low temperature and high temperature heat release, negative temperature coefficient behavior, etc. Identifying all the individual molecules in a fuel along with their composition using methods like DHA is expensive and time consuming, especially during post processing of the obtained data. Expressing the fuel’s composition in the form of functional groups is an alternate way of presenting the fuel’s complex molecular information. Hydrocarbon fuels like gasoline are usually made up of as many as six or seven functional groups [8]; this methodology helps to condense the diverse molecular composition of the fuel into a short and concise format.

Allen et al. [9] were the first to characterize a sample of coal-derived liquid using functional groups. A functional group is a collection of atoms in a larger molecule that governs its properties. It has been shown that physical properties such as viscosity and density [10], thermochemical properties such as heat of formation [11] and specific heat capacity [12], and combustion properties such as octane number (ON) [13], cetane number [14], flash point [15], flame speed [16], etc., are dependent on the groups in the fuel. Abdul Jameel et al. [8] expressed the standard FACE gasoline fuels using six functional groups as follows: paraffinic CH_3_, CH_2_ and CH groups, olefinic CH=CH_2_ groups, naphthenic CH-CH_2_ groups, and aromatic C-CH groups. These six groups were set as a matching criterion for the formulation of surrogates for FACE gasolines. This methodology, referred to as the minimalist functional group (MFG) approach [8,17,18], was then later extended to jet fuel and diesel. The RON and MON of gasoline–ethanol mixtures have also been predicted using the aforementioned functional groups along with the hydroxyl (OH) group [19]. Dooley et al. [20] employed the methyl (CH_3_), methylene (CH_2_) and benzyl (C_6_H_5_CH_2_) groups to express the composition of jet fuel surrogates in the form of the three functional groups and found that these groups dictate the formation of the radical pool. Sumathi et al. [21,22] used the alkyl and free radical groups to predict the reaction rates of various reaction sets appearing in a number of kinetic models by using the functional group additivity approach. A number of other studies [23,24,25] have also been reported that have shown the dependence of fuel properties on the constituent functional groups.

^1^H-NMR spectroscopy is ideally suited to the identification and quantification of ^1^H atom types in a complex mixture like petroleum fuel. The various groups present in the sample produce distinct and characteristic peaks in the ^1^H-NMR spectra. In addition, hydrocarbon functional groups that exhibit similar molecular structures also give rise to separate signals, which is convenient for identifying and quantifying them. ^1^H-NMR spectroscopy is a non-destructive analytical technique that produces high-resolution data, which is quick and consistently reproducible. Gas chromatography (GC)-based DHA analysis [6] is a more time consuming technique, whereas ^1^H-NMR spectra can be collected in a matter of minutes. ^1^H-NMR spectroscopy is well suited to high-throughput labs thanks to the ability to turn out a large number of samples in a rapid manner. The objective of the present work is to identify and quantify the functional groups present in a gasoline sample using ^1^H-NMR spectroscopy and to use the obtained functional groups to predict a number of fuel properties.

## 2. Experimental Method

**Figure 1:**
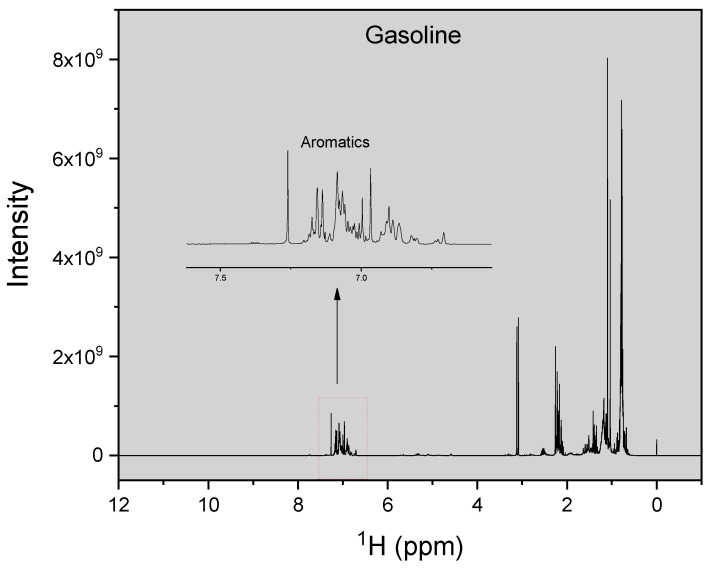
^1^H-NMR spectra of the gasoline sample.

A premium gasoline sample was collected from a commercial gas station in Dhahran, Saudi Arabia and was then refrigerated in order to prevent the escape of the volatile components during storage. For the ^1^H-NMR measurements, 100 µL of the refrigerated gasoline sample was mixed with 2 mL of deuterated chloroform (CDCl_3_), which was used as a solvent. The mixture was brought to ambient temperature before the measurements. The advantage of using CDCl_3_ is that there is no solvent peak in the ^1^H-NMR spectra due to the absence of H atoms in the solvent. The gasoline sample was completely dissolved in the solvent upon visual inspection. 1 mL of the dissolved mixture was then transferred into standard 5 mm NMR tubes. The ^1^H-NMR measurements were made at 298 K using a 400 MHz Bruker spectrometer. A recycle delay of 5 s was used and 128 scans were collected. The pulse sequence used was 1D 90°, along with the ‘zg’ standard program in the Bruker library. Tetramethylsilane (TMS), which has a strong and sharp resonance line, was used as the internal standard. The free induction decay (FID) signal was collected by using a spectral width of 14,098 Hz, which was digitized into 64 K data points. MestreNova software was used for processing the ^1^H-NMR spectra, which were obtained after Fourier transformation using a line broadening of 1 Hz. The spectra were manually phase corrected, then the baseline was corrected using the Bernstein polynomial technique pre-programed in the software. The ^1^H-NMR spectra for the gasoline sample are shown in Figure 1. A total of three samples were measured; the ^1^H-NMR spectra of the other two samples are given in the Supplementary Material, as these additional samples have similar compositions.

## 3. Functional Group Analysis

^1^H-NMR spectra are shown with chemical shift in the x axis and signal intensity in the y axis. Chemical shift is defined as the resonant frequency of a nucleus in the presence of a magnetic field, with the usual range used for ^1^H-NMR spectra are between 0–12 ppm. The internal standard, TMS, is assigned a chemical shift of 0 ppm. Molecular structure elucidation is based on the position and number of characteristic chemical shifts, from which the structure and atom type is deduced. In complex mixtures such as petroleum gasoline there is some overlap in the peaks, and thus a range for the occurrence of the functional groups is usually specified. The ^1^H-NMR chemical shifts used for identifying the groups were taken from Abdul Jameel et al. [8] and are presented in Table 1. The range between 1.39–2.05 ppm may give rise to overlapping peaks from the CH and CH_2_ groups present in iso-paraffins and naphthenes [26]. There is uncertainty associated with the calculation of these groups from the spectra. However, functional group comparisons using both the above methodology and those calculated from detailed hydrocarbon analysis have resulted in good comparison [14], and therefore the above methodology is warranted for use. Slight variations of the ranges discussed in Table 1 have also been discussed in the literature [27,28].

**Figure 2:**
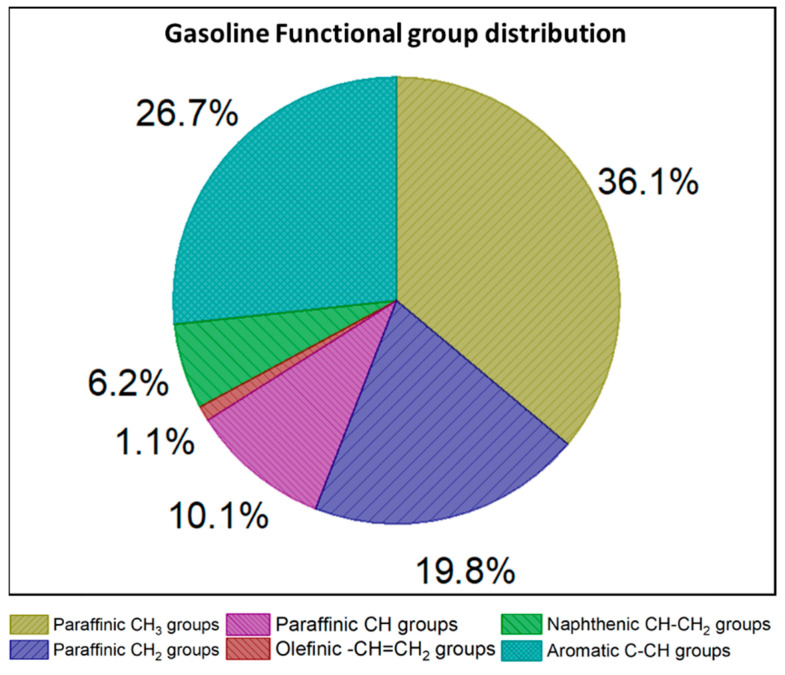
Mass % of the functional groups present in the gasoline sample.

The composition of the H type (in mol%) is obtained by dividing the integral intensity of the particular region (i.e., aromatic hydrogen is represented by ‘a’ and so forth) by the sum of the integral intensities (S). The resultant ratio is then converted to a percentage basis. The mass percentage of the hydrocarbon functional groups (the combination of carbon and hydrogen atoms) is evaluated by including the carbon atom in the functional group, along with its molecular weight. As an example, the mass percentage of the paraffinic CH_2_ group is calculated by multiplying the ^1^H types (from ‘d’ and ‘h’) with its molecular weight (i.e., 14). The formulae used for calculating the mass percentage of the various functional group in the gasoline sample are shown in Table 2. The molecular weight corresponding to the olefinic and naphthenic groups was calculated (as 13.5) by making an assumption that the H atoms present in the sample were equally divided between CH and CH_2_ groups. The mass of quaternary aromatic carbons was also considered to be included in the aromatic C-CH groups by considering the three paraffinic groups present in the alpha position of the aromatic ring. The mass percentages of the groups present in the gasoline sample are shown in Figure 2. Paraffinic CH_3_ groups appears to be largest class in the sample, followed by aromatic groups, while olefins have the lowest concentration of any chemical class.

## 4. Property Prediction

### 4.1. Octane Number (ON)

**Figure 3:**
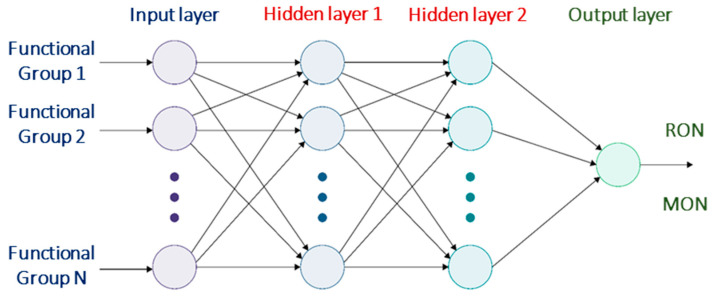
Architecture of a neural network model foe RON and MON prediction.

For gasolines, ON is used to quantify the fuel’s ability to resist knocking, i.e., premature combustion that occurs prior to the spark ignition on account of compression of the air–fuel mixture inside the engine. RON and MON are two octane ratings which are experimentally quantified using a co-operative fuel research (CFR) engine. The standard ASTM D2699-16 [29] is employed for measuring RON, whereas ASTM D2700-16a [30] is used for MON. Experimental measurements of RON and MON are very expensive, involve many man-hours, require large volumes of reference fuels, and necessitate skilled operators and sophisticated instrumentation, thereby warranting the need for predictive models. There are many works [6,31,32,33] present in literature that deal with the development of models to predict the ON of pure components and blends using group contribution and structure-property relationships. Abdul Jameel et al. [19] developed a neural network model that predicts the RON and MON of gasoline-ethanol fuels by using seven functional groups that make up most of the fuel. The model was developed by using 281 fuels comprised of pure compounds and blends. A neural network model has an input layer into which the amounts of the functional groups (in wt%) are fed, one or more hidden layers where the calculations are performed, and an output layer where the target (RON/MON) is arrived at as the output. The general architecture of a neural network model is shown in Figure 3. Two more input features, namely molecular weight and branching index, were also employed as additional inputs to the neural network model for predicting RON and MON. The hydrocarbon functional groups obtained in the present study were fed into the model for predicting the RON and MON of the gasoline sample; these values are reported in Table 3. The ANN model [19] used for predicting RON had two hidden layers, 540 nodes in the first layer and 314 nodes in the second layer. The model for RON also had two layers, with 340 and 603 nodes in the first and second layer. The predicted RON value of 95.8 of the present premium gasoline sample is consistent with the expected value of 95 as advertised by the gas station. The gasoline has a high-octane sensitivity (RON-MON) [34] of 13.3, which is due to the large content of aromatic groups. Octane sensitivity is relevant to resistance against knocking inside the SI engine, especially at high loads.

### 4.2. Derived Cetane Number (DCN)

The quality of a diesel fuel used to run a compression-ignited (CI) diesel engine is expressed in the form of cetane rating or cetane number (CN), which is similar to the ON used for gasoline. The fraction of the time passed between diesel injection and subsequent combustion inside the CI diesel engine is called the IDT. The CN of the fuel is then calculated from the numerical value of the IDT. There are three standard methods used for measurement of the CN (also termed ignition quality) of diesel, namely: ASTM D613 [35], which uses a CFR engine; ASTM D7170 [36]. which employs a fuel ignition tester; and ASTM D6890 [37], which uses an ignition quality tester (IQT) to measure the IDT of the fuel, from which CN is calculated. The CN calculated from the ASTM D6980 standard is called the DCN, which is functionally similar to the CN. As is the case with RON and MON measurements, experimental measurement of CN/DCN is expensive, and there are multiple studies [33,38,39] reported in the literature that have developed models for CN/DCN prediction. Recently, an artificial neural network-based technique [40] was developed for predicting of the DCN of fuels containing oxygenated classes like alcohols and ethers by using the functional groups as input parameters. The DCN prediction model was developed by using 499 fuels, and the model consisted of two hidden layers with 442 nodes in the first layer followed by 290 nodes in the second. The DCN of the present gasoline sample predicted using the neural network model is presented in Table 3. It is noteworthy to mention that measuring and predicting the DCN of gasoline fuels has become relevant due to the advent of advanced engine technologies such as gasoline compression ignition (GCI) engines [41]. As CN and ON are inversely related, the predicted DCN of the gasoline sample was low at a value of 17.5, as expected. A typical diesel fuel possesses a CN in the vicinity of 40 to 50, whereas premium diesel fuels have a higher CN, around 55–60.

### 4.3. Sooting Propensity

Soot consists of carbonaceous particles that are emitted due to incomplete combustion of hydrocarbon fuels, and is classified as one of the most detrimental sources of global warming after CO_2_ emissions. Soot particles with sizes of less than 2.5 micrometers are also known to cause health issues such as bronchitis, asthma, cancer, etc. Though gasoline fueled SI engines produce less soot than diesel-fueled compression-ignited (CI) engines, the net impact of soot from SI engines is higher due to the use of particulate matter (PM) filters in diesel exhaust [42]. Empirical methods like TSI [43] and YSI [44] have been employed by researchers to measure the propensity of a fuel to produce soot upon combustion. TSI is experimentally measured in a smoke point (SP) lamp from the height of a smoke free laminar flame, from which TSI is evaluated [43]. YSI is measured by employing a non-premixed methane diffusion flame where the fuel to be tested is doped in ppm-level concentrations [44]. The maximum soot volume fraction is then determined, from which YSI is calculated. Recently a functional group-based neural network model [45] was proposed for prediction of the TSI and YSI of oxygenated fuels. The YSI model was developed by using 265 neat compounds with hidden layers consisting of 25 nodes in each layer. The TSI and YSI values of the present gasoline sample is presented in Table 3. Predictions of TSI and YSI were slightly greater than expected due to the large amount of aromatic C-CH groups at 26.7%. The predicted properties of the additional samples are provided in the supplementary material. These additional samples have similar properties to the gasoline discussed here, due to similar composition and functional groups.

## 5. Conclusions

In the present investigation, ^1^H-NMR spectroscopy was used for the identification and quantification of hydrocarbon functional groups in a premium gasoline sample. Six functional groups were identified and quantified; their values in mass percentage are as follows: paraffinic CH_3_ group (36.1%); paraffinic CH_2_ groups (19.8%); paraffinic CH group (10.1%); olefinic-CH=CH_2_ groups (1.1%); naphthenic CH-CH_2_ groups (6.2%); and aromatic C-CH groups (26.7%). The predicted values for the RON and MON of the gasoline sample, obtained using a neural network model with functional group inputs, were calculated as 95.8 and 82.5, respectively. The high-octane sensitivity of the fuel was expected due to the large aromatic content. The predicted DCN of the gasoline was 17.5; this low value was expected, as ON and CN are inversely proportional to each other. The predicted TSI and YSI were 21.7 and 76, respectively. The sooting tendency of the present gasoline sample is on the higher side due to the high content of aromatics, which act as soot precursors. Identifying and quantifying the fuel functional groups presents a robust and much easier alternative to the characterization of real petroleum fuels. From the functional groups, a number of physical, chemical and combustion properties can be predicted.

## Figures and Tables

**Table 1:** Characteristic ^1^H-NMR chemical shifts for identifying the functional groups.

H Atom	Chemical Shift (ppm)	Integral Intensity
aromatic C-CH	6.42–8.99	a
olefinic CH=CH_2_	4.50–6.42	b
paraffinic CH group in alpha position to aromatic ring	2.88–3.40	c
paraffinic CH_2_ group in alpha position to aromatic ring	2.64–2.88	d
paraffinic CH_3_ group in alpha position to aromatic ring	2.04–2.64	e
naphthenic CH-CH_2_ group	1.57–1.96	f
paraffinic CH groups	1.39–1.57	g
paraffinic CH_2_ group	0.94–1.39	h
paraffinic CH_3_ group	0.25–0.94	i
Sum (a + b + c + d + e + f + g + h + i)		S

**Table 2:** Formulae for quantifying the hydrocarbon functional groups by mass %.

Functional Group	H Type (mol %)	Mass (Arbitrary Unit)	Mass (%)
Paraffinic CH_3_	$H_{P_{CH_{3}}}\;=\;\frac{\left(i\;+\;e\right)}{S}\;*\;100$	$U_{P_{CH_{3}}}\;=\;\frac{H_{P_{CH_{3}}}\;*\;15}{3}$	$M_{P_{CH_{3}}}\;=\;\frac{U_{P_{CH_{3}}}\;*\;100}{U_{P_{CH_{3}}}\;+\;U_{P_{CH_{2}}}\;+\;U_{P_{CH}}\;+\;U_{O_{CH.CH_{2}}}\;+\;U_{N_{CH.CH_{2}}\;}+\;U_{A_{C.CH}}\;}$
Paraffinic CH_2_	$H_{P_{CH_{2}}}\;=\;\frac{\left(h\;+\;d\right)}{S}\;*\;100$	$U_{P_{CH_{2}}}\;=\;\frac{H_{P_{CH_{2}}}\;*\;14}{2}$	$M_{P_{CH_{2}}}=\frac{U_{P_{CH_{2}}}\;*\;100}{U_{P_{CH_{3}}}\;+\;U_{P_{CH_{2}}}\;+\;U_{P_{CH}}\;+\;U_{O_{CH.CH_{2}}}\;+\;U_{N_{CH.CH_{2}}\;}+\;U_{A_{C.CH}}}$
Paraffinic CH	$H_{P_{CH}}=\frac{\left(g\;+\;c\right)}{S}\;*\;100$	$U_{P_{CH}}\;=\;\frac{H_{P_{CH}}\;*\;13}{1}$	$M_{P_{CH}}\;=\;\frac{U_{P_{CH}}\;*\;100}{U_{P_{CH_{3}}}\;+\;U_{P_{CH_{2}}}\;+\;U_{P_{CH}}\;+\;U_{O_{CH.CH_{2}}}\;+\;U_{N_{CH.CH_{2}}\;}\;+\;U_{A_{C.CH}}}$
Olefinic CH=CH_2_	$H_{O_{CH.CH_{2}}}\;=\;\frac{b}{S}\;*\;100$	$U_{O_{CH.CH_{2}}}\;=\;\frac{H_{O_{CH.CH_{2}}}\;*\;13.5}{1.5}$	$M_{O_{CH.CH_{2}}}\;=\;\frac{U_{O_{CH.CH_{2}}}\;*\;100}{U_{P_{CH_{3}}}\;+\;U_{P_{CH_{2}}}\;+\;U_{P_{CH}}\;+\;U_{O_{CH.CH_{2}}}\;+\;U_{N_{CH.CH_{2}}\;}+\;U_{A_{C.CH}}}$
Naphthenic CH-CH_2_	$H_{N_{CH.CH_{2}}}\;=\;\frac{f}{S}\;*\;100$	$U_{N_{CH.CH_{2}}}\;=\;\frac{H_{N_{CH.CH_{2}}}\;*\;13.5}{1.5}$	$M_{N_{CH.CH_{2}}}\;=\;\frac{U_{N_{CH.CH_{2}}}\;*\;100}{U_{P_{CH_{3}}}\;+\;U_{P_{CH_{2}}}\;+\;U_{P_{CH}}\;+\;U_{O_{CH.CH_{2}}}\;+\;U_{N_{CH.CH_{2}}\;}+\;U_{A_{C.CH}}}$
α-CH	$H_{\alpha -\mathrm{CH}}\;=\;\frac{c}{S}\;*\;100$		
α-CH_2_	$H_{\alpha -{\mathrm{CH}}_{2}}\;=\;\frac{d}{S}\;*\;100$		
α-CH_3_	$H_{\alpha -{\mathrm{CH}}_{3}}\;=\;\frac{e}{S}\;*\;100$		
*Aromatic C-CH*	$H_{A_{C.CH}}\;=\;\frac{a}{S}\;*\;100$	$U_{A_{C.CH}}\;=\;\frac{H_{A_{C.CH}}\;*\;13}{1}\;+\;\frac{H_{\alpha -CH}\;*\;13}{1}\;+\;\frac{H_{\alpha -CH_{2}}\;*\;14}{2}\;+\;\frac{H_{\alpha -CH_{3}}\;*\;15}{3}$	$M_{A_{C.CH}}\;=\;\frac{U_{A_{C.CH}}\;*\;100}{U_{P_{CH_{3}}}\;+\;U_{P_{CH_{2}}}\;+\;U_{P_{CH}}\;+\;U_{O_{CH.CH_{2}}}\;+\;U_{N_{CH.CH_{2}}\;}+\;U_{A_{C.CH}}}$

**Table 3:** Predicted fuel properties from the functional groups.

Property	Value (No Unit)
RON	95.8
MON	82.5
DCN	17.5
TSI	21.7
YSI	76

## Supplementary Materials

The following are available online, Figure S1: ^1^H-NMR spectra of the other gasoline samples measured, Table S1: Predicted fuel properties for additional samples

## Sample Availability

Samples of the compounds are not available from the authors.
