# Supplementary material for: Identification and Quantification of Hydrocarbon Functional Groups in Gasoline Using 1H-NMR Spectroscopy for Property Prediction
Source: Molecules. 2021 Nov 19;26(22):6989. doi: 10.3390/molecules26226989 (PMC8622857; doi:10.3390/molecules26226989)
Supplement: Supplementary file 1 [file molecules-26-06989-s001.zip › molecules-1450718-supplementary.pdf]

Supporting Information

Identification and Quantification of Hydrocarbon Functional Groups in Gasoline using 1H NMR Spectroscopy for Property Prediction

Abdul Gani Abdul Jameel [1, 2*]

[1] Department of Chemical Engineering, King Fahd University of Petroleum & Minerals, Dhahran 31261, Saudi Arabia

[2] Center for Refining & Advanced Chemicals, King Fahd University of Petroleum & Minerals, Dhahran 31261, Saudi Arabia

Correspondence Abdul Gani Abdul Jameel < a.abduljameel@kfupm.edu.sa >

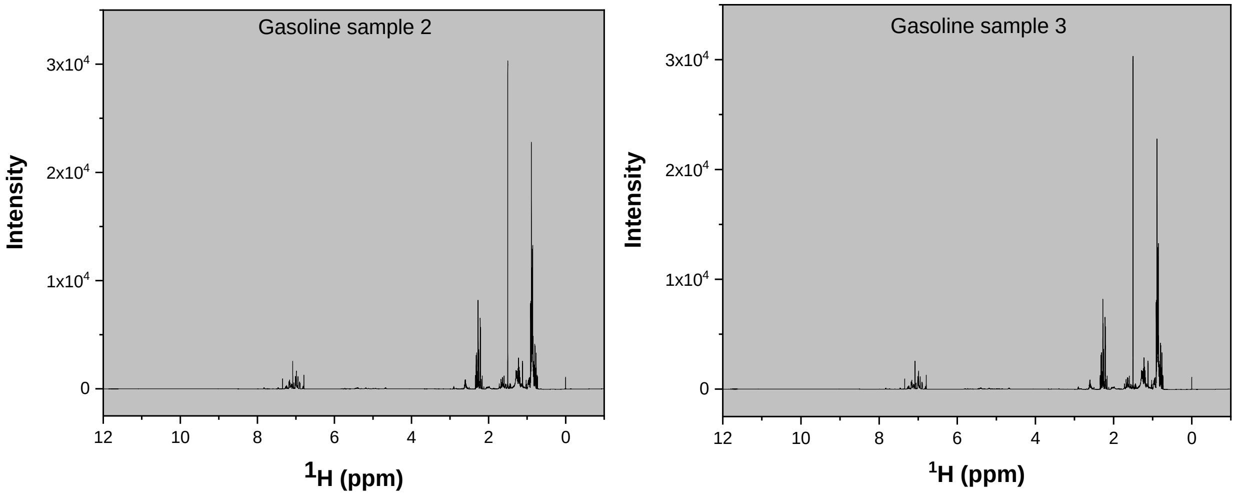

Figure S1. ^{1}H NMR spectra of the other gasoline samples measured

Table S1. Predicted fuel properties for additional samples

Property	Sample 2 (No unit)	Sample 3 (No unit)
RON	95.0	96.1
MON	82.0	82.7
DCN	17.8	17.2
TSI	22.1	21.2
YSI	76.4	75.8
